# What is the best cutoff point of leukocyte esterase for diagnosis of periprosthetic joint infections? a systematic review and meta-analysis

**DOI:** 10.1186/s42836-025-00325-y

**Published:** 2025-08-05

**Authors:** Mohammad Poursalehian, Ali Soltani Farsani, Pouya Tabatabaei Irani, Mohammad Ayati Firoozabadi, Javad Parvizi, SM Javad Mortazavi

**Affiliations:** 1 Joint Reconstruction Research Center, Tehran University of Medical Sciences, Tehran, 1419733141 Iran; 2 Department of Orthopedic Surgery, Imam Khomeini Hospital Complex, Tehran University of Medical Sciences, Tehran, 1419733141 Iran; 3 Department of Orthopedics and Traumatology, Acibadem Mehmet Ali Aydınlar University, Istanbul, 34752 Turkey; 4 International Joint Centre (IJC), Acibadem Maslak Hospital, Istanbul, 34457 Turkey

**Keywords:** Periprosthetic joint infection, Total joint arthroplasty, Leucocyte esterase test, Diagnostic accuracy, Centrifugation, Biomarkers

## Abstract

**Background:**

Periprosthetic joint infection (PJI) is a significant complication following total joint arthroplasty that demands rapid, accurate diagnosis. The leukocyte esterase (LE) test shows promise, but studies vary in cut-off values and omit the centrifugation’s effect. In this study, we assessed the sensitivity and specificity of the LE test across different cut-off values, both with and without centrifugation. We aimed to identify the optimal threshold for diagnosing PJI and to compare its diagnostic odds ratio (DOR) to those of biomarkers recommended by the International Consensus Meeting (ICM).

**Methods:**

A comprehensive literature search was performed in PubMed, Scopus, Web of Science, and Embase up to May 2024. Studies were included if they evaluated the diagnostic accuracy of LE for PJI in TJA and provided sufficient data for constructing 2 × 2 contingency tables. Data extraction and quality assessment were independently conducted by two reviewers using a standardized form and the QUADAS-2 tool. Statistical analysis involved pooling data using a bivariate random-effects model and constructing summarized receiver operating characteristic (sROC) curves.

**Results:**

Out of 2195 records, 26 studies involving 4,206 joints (1,282 with PJI) were included. The optimal LE cut-off point without centrifugation was 3 + , yielding a sensitivity of 0.877, a specificity of 0.957, and a DOR of 159.2. With centrifugation, a 2 + cut-off provided a sensitivity of 0.899, a specificity of 0.924, and a DOR of 108.6. Direct comparison with other biomarkers indicated that polymorphonuclear neutrophils percentage (PMN%), white cell count (WCC), and alpha defensin (AD) had a slightly higher diagnostic odds ratio and Youden index than LE. Direct comparison with other biomarkers also indicated that erythrocyte sedimentation rate (ESR), serum C-reactive protein (CRP), synovial CRP, and D-dimer had lower DOR and Youden index than LE.

**Conclusions:**

The LE test is an effective diagnostic tool for PJI. Adopting a 3 + cut-off point without centrifugation and a 2 + one with centrifugation optimizes diagnostic accuracy.

**Supplementary Information:**

The online version contains supplementary material available at 10.1186/s42836-025-00325-y.

## Introduction

Periprosthetic joint infection (PJI) is a significant complication following total joint arthroplasty (TJA) [1], and is one of the most common causes leading to revision surgery [2]. A significant portion of all the studies related to TJA focus on PJI, making it the most extensively researched aspect of TJA [3, 4]. Between 1 and 2% of patients who receive joint arthroplasty may experience PJI [5, 6]. The symptoms of PJI are usually not specific [7]. Therefore, the diagnosis of PJI can be challenging. Despite advances in diagnostic techniques, there remains a need for rapid, reliable, and cost-effective methods to identify PJI.

The leucocyte esterase (LE) test was initially developed to detect urinary tract infections [8]. But it gained attention as a potential diagnostic tool for PJI [9–11]. LE is an enzyme produced by activated neutrophils, and its presence in synovial fluid can indicate infection [12]. In connection with the number of neutrophils present, the colorimetric scale is graded as zero (negative test), mild (1 +), moderate (2 +), and high (3 +) [13]. The LE test has several advantages, including ease of use, rapid results, and low cost, with an estimated cost of $0.17 per test [14, 15]. However, a significant challenge in employing the LE test for diagnosing PJI is establishing the optimal cut-off threshold and addressing its inherent operator dependency [16, 17].

The current diagnostic criteria for PJI, established by the Musculoskeletal Infection Society (MSIS) [18] and the International Consensus Meeting (ICM) [19], include a combination of clinical, serological, and microbiological parameters. Among these, the LE test is one of the criteria. Several studies have investigated the usefulness of the LE test for PJI diagnosis [20–22]. However, there is variability in the reported cut-off points, leading to inconsistencies in its clinical application [23–25]. Some studies suggest that a reading of 2+ (moderate) may have the most accuracy [20, 26], while others suggest a reading of 3 + (high) [24, 25].

To date, no systematic review has thoroughly examined the optimal cut-off point for LE in diagnosing PJI, nor has the combined effect of centrifugation and varying cut-off thresholds been comprehensively evaluated. In this study, we will first investigate the sensitivity and specificity of the LE test across different cut-off levels, followed by an assessment of how centrifugation of synovial fluid samples affects test accuracy. By integrating these findings, we aim to identify the most accurate LE cut-off point for diagnosing PJI. Additionally, we will directly compare the sensitivity, specificity, and DOR of the LE test with those of other serum and synovial biomarkers recommended by the ICM definition of PJI.

## Methods

We carried out the current systematic review and reported the findings by the standards of the Preferred Reporting Items for Systematic Reviews and Meta-Analyses (PRISMA) [27]. This systematic review’s protocol was registered in PROSPERO (CRD42024511117).

### Search strategy

A comprehensive literature search was conducted in PubMed, Scopus, Web of Science, and Embase up to May 2024. The keywords used were “Leukocyte Esterase” OR “Leucocyte Esterase”. No language restrictions were imposed. Additional studies were identified by manually searching the references of the included articles.

### Inclusion and exclusion criteria

Studies were included if they evaluated the diagnostic accuracy of the LE test in detecting PJI among patients undergoing TJA and provided adequate data to construct 2 × 2 contingency tables for sensitivity and specificity analysis. Only studies employing a reference standard other than the LE test, such as histological analysis or periprosthetic tissue culture, were considered to ensure methodological rigor to confirm PJI. Studies were excluded if they were case reports, letters, editorials, reviews, conference abstracts, or animal/laboratory research. Moreover, studies that assessed LE in contexts unrelated to PJI (such as osteomyelitis), reported duplicated data from previously published findings, or were published in languages other than English were also excluded.

### Data extraction and quality assessment

Two independent reviewers (MP, ASF) extracted data using a standardized form. In cases of conflict, a third senior reviewer (SMJM) resolved the dispute. Information collected included study design, patient characteristics, diagnostic tests evaluated, and measures of diagnostic accuracy. The quality of the studies was assessed using the QUADAS-2 tool [28]. The QUADAS-2 tool consists of four key domains (i.e., patient selection, index test, reference standard, and flow and timing). The risk of bias was assessed in each domain, and concerns about applicability were evaluated in the first three domains with signaling questions. These questions were answered with “yes” for a low risk of bias/concerns, “no” for a high risk of bias/concerns, or “unclear” when relevant information was not provided.

### Statistical analysis

We categorized the study results based on their reported cutoff points and whether centrifugation was performed before conducting the strip test. Given that different LE test strips were utilized across the studies, each with its distinct grading scale, we standardized the LE results to the Combur-Test® strip (Roche Diagnostics GmbH, Mannheim, Germany) to ensure consistency and comparability [29]. To achieve this standardization, we aligned the LE test cutoff values with their corresponding LE concentration per microliter (µL), as defined by the Combur-Test® strip. The standardized values derived from the Combur-Test® were as follows: negative = 0 LuE/µL, 1 +  = 10–25 LuE/µL, 2 +  = 75 LuE/µL, and 3 +  = 500 LuE/µL.

Data were pooled using a bivariate random-effects model. We calculated summary estimates of sensitivity, specificity, positive and negative likelihood ratios (positive likelihood ratio (PLR) and negative likelihood ratio (NLR)), and DOR. The bivariate model employs a random-effects approach, and the statistical properties of the bivariate model are suited to performing diagnostic meta-analyses. In addition, summarized receiver operating characteristic (sROC) curves were constructed. All analyses were performed using MetaDTA v2 [30].

## Results

### Search results

**Fig. 1:**
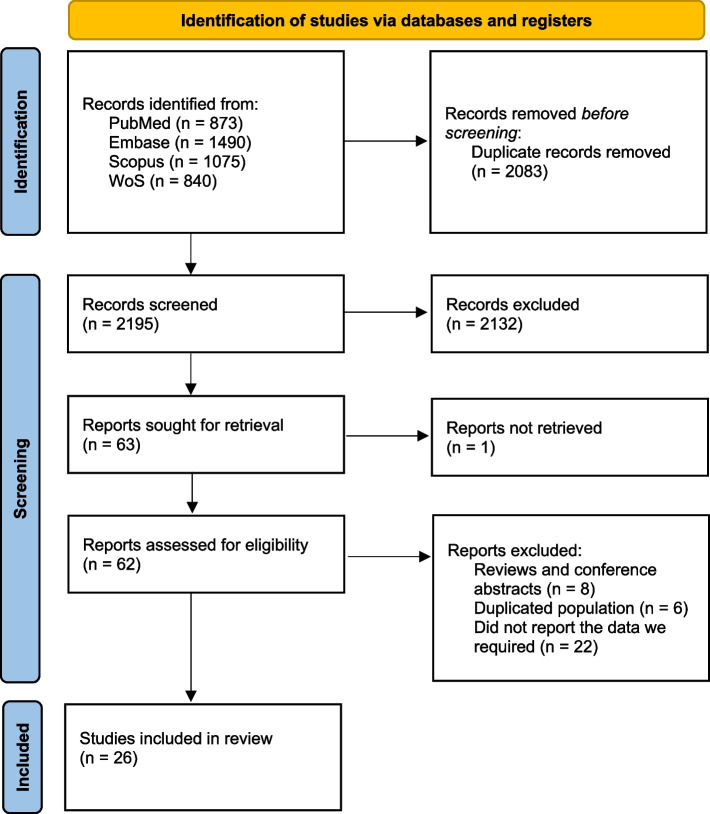
PRISMA flow diagram of included studies

A total of 2195 records were identified by searching databases and removing duplicates. After the initial screening of titles and abstracts, 62 articles were further assessed by scrutinizing the full texts against the predesigned criteria, and eventually, 26 articles were included in the quantitative analysis. Selection processes for eligible studies are depicted in Fig. 1.

### Study characteristics

Thirteen studies were prospective, and thirteen studies were retrospective. All studies were cohort studies. Twenty-six studies involving 4,206 joints (1,282 joints with PJI) explored the diagnostic accuracy of LE. The mean ages of included patients ranged from 60.3 to 71.0 years, and the proportion of males ranged from 21.7% to 60.9%. In addition, six studies assessed the accuracy of erythrocyte sedimentation rate (ESR) [20, 26, 31–34], eight studies evaluated the accuracy of serum C-reactive protein (CRP) [20, 26, 29, 31–35], four studies evaluated the accuracy of synovial CRP [20, 23, 26, 36], eight studies examined the accuracy of synovial White Cell Count (WCC) [20, 23, 26, 29, 31–33, 35], seven studies analyzed the accuracy of synovial polymorphonuclear neutrophil percentage (PMN%) [20, 26, 29, 31–33, 35], nine studies investigated the accuracy of alpha defensin (AD) [20–23, 26, 29, 33, 35, 37], and two studies investigated the accuracy of D-dimer [20, 33]. The main characteristics of the included studies are summarized in Table 1.

**Table 1:** Characteristics of included studies

Citation	Design of study*	Brand of Strip Test, Company, N blocks	Dates of study	Centrifugation	No. of patients	Gold standard of diagnosing PJI	Cut-off for LE test **
Parvizi et al. [11]	P	Chemstrip 7, Roche, 4 blocks	2007–2010	No	125	Similar to Musculoskeletal Infection Society (MSIS) 2011	2 +/3 +
Guenther et al. [38]	P	Combur 2, Roche, 4 blocks	NA	No	364	Intra-articular culture	2 +
Shafafy et al. [39]	P	Multisix 8, Siemens, 5 blocks	2012–2013	No	105	Infectious Diseases Society of America (IDSA) 2013	2 +/3 +
Colvin et al. [40]	R	Chemstrip 7, Roche, 4 blocks	2013–2014	No	57	Institutional criteria	3 +
Deirmengian et al. [41]	P	Chemstrip 7, Roche, 4 blocks	2012	No	46	MSIS 2011	3 +
De Vecchi et al. [36]	P	ECS, Dirui, 4 blocks	2014–2015	Yes	129	International Consensus Meeting (ICM) 2013	2 +
Tischler et al. [31]	P	NR, NR, 4 blocks	2010–2015	Yes	61	MSIS 2011 & ICM 2013	3 +
Ruangsomboon et al. [42]	R	Chemstrip 10, Roche, 4 blocks	2015–2016	Yes	46	ICM 2013	2 +
Koh et al. [43]	P	3 different strip tests (Aution 11, Clinitek 500, and Urisys 2400), all 4 blocks	2013–2015	Yes	60	ICM 2013	2 +/3 +
Shahi et al. [32]	R	NR, NR, 4 blocks	2000–2015	No	659	MSIS 2011 (without minor criteria)	2 +
Li et al. [24]	P	Aution 10, Arkray, 5 blocks	2016–2017	Both method	133	ICM 2013	2 +/3 +
Li et al. [25]	P	Aution 10, Arkray, 5 blocks	2014–2016	No	204	MSIS 2011	2 +/3 +
De Vecchi et al. [23]	P	ECS, Dirui, 5 blocks	2015–2017	Yes	66	ICM 2013	2 +/3 +
Di Benedetoo et al. [44]	R	Chemstrip 7, Roche, 4 blocks	2016–2019	No	51	ICM 2013	2 +
Zagra et al. [10]	R	ECS, Dirui, 4 blocks	2015–2017	Yes	119	ICM 2013	2 +
Sharma et al. [26]	R	Multisix 10, Siemens, 5 blocks, and Chemstrip 10, Roche, 4 blocks	2000–2018	Yes	107	MSIS 2011	2 +
Yu et al. [22]	R	Aution 10, Arkray, 5 blocks	2015–2018	Both method	130	Similar to ICM 2013	2 +/3 +
Levent et al. [29]	R	Combur 10, Roche, 4 blocks	2015–2017	No	260	ICM 2013 & 2018	2 +
Chisari et al. [20]	R	Chemstrip 7, Roche, 4 blocks	2009–2019	Yes	259	ICM 2018	2 +/3 +
Shohat et al. [21]	R	NR, Roche, 4 blocks	2013–2019	Yes	122	ICM 2018	3 +
Kuo et al. [33]	R	AUCA, Siemens, 5 blocks	2018–2019	No	76	ICM 2018	2 +
Haertle et al. [45]	R	Combur, Roche, 4 blocks	2014–2017	Yes	145	ICM 2018	2 +
Logoluso et al. [34]	R	ECS, Dirui, 4 blocks	2015–2020	Yes	79	ICM 2018	2 +
Grzelecki et al. [46]	R	Aution 10, Arkray, 5 blocks, and BM 10, BioMaxima, 5 blocks	2021–2022	Yes	110	ICM 2018	2 +
Grunwald et al [37]	P	Combur 10, Roche, 4 blocks	2018–2022	Yes	249	European Bone and Joint Infection Society (EBJIS) 2021	1 +
Burchette et al. [35]	R	NR, NR, 4 blocks	2014–2018	No	362	ICM 2018	2 +

^*^P for prospective and R for retrospective, **All the values converted to the Combur urine test strip grading

### Gold standard for diagnosing PJI

The studies included in this analysis employed varying criteria for diagnosing PJI (Table 1). The first widely accepted definition of PJI was established by the Musculoskeletal Infection Society (MSIS) in 2011, offering a structured diagnostic approach [47]. In 2013, the inaugural International Consensus Meeting (ICM) on PJI refined these criteria, and subsequent updates, notably the ICM 2018, further enhanced the diagnostic framework by introducing additional biomarkers [18, 48]. The most recent definition, released by the European Bone and Joint Infection Society (EBJIS) in 2021, proposed a three-tiered classification system for PJI, distinguishing between confirmed, likely, and unlikely infections [49]. However, the core diagnostic principles remained consistent across various criteria; newer iterations have become more comprehensive, incorporating novel biomarkers and adjusting cutoff values for existing tests. Table 2 summarizes the different criteria associated with PJI.

**Table 2:** Different definitions that have been introduced for diagnosing periprosthetic joint infection (PJI)

Definition of PJI	Major Criteria	Minor Criteria	Diagnostic Threshold
Musculoskeletal Infection Society (MSIS) 2011[47]	1- A sinus tract communicating with the prosthesis, 2- A pathogen is isolated by culture from two separate tissues or fluid samples obtained from the affected prosthetic joint	a. Elevated Serum erythrocyte sedimentation rate (ESR) and C-reactive protein (CRP) (ESR > 30 mm/h; CRP > 10 mg/L), b. Elevated synovial fluid white blood cell (WBC) count (> 3000), c. Elevated synovial fluid neutrophil percentage (> 65%), d. Presence of purulence in the affected joint, e. Isolation of a microorganism in one periprosthetic tissue or fluid culture, f. Greater than 5 neutrophils per high-powered field (HPF) in 5 HPFs observed from histological analysis of periprosthetic tissue (× 400)	One major criterion or four of six minor criteria
International Consensus Meeting (ICM) on PJI 2013[48]	1- Two positive periprosthetic cultures with phenotypically identical organisms, 2- A sinus tract communicating with the joint	a. ESR > 30 mm/h after 90 days from index surgery, and serum CRP level > 10.0 mg/dL within 90 days after index surgery or 1.0 mg/dL after 90 days from index surgery; b. Synovial fluid WBC count > 10,000 cells/μL within 90 days after the index surgery or > 3,000 cells/μL after 90 days from the index surgery; c. Synovial fluid polymorphonuclear neutrophils percentage (PMN%) > 90% within 90 days of the index surgery or > 80% after 90 days from index surgery; d. Isolation of microorganisms from the preoperative joint fluid or intraoperative tissue cultures; e. More than five neutrophils per HPF in five HPFs (× 400)	One major criterion or three of five minor criteria
Infectious Diseases Society of America (IDSA) 2013[50]	1- Sinus tract communicating with the prosthesis, 2- Two or more positive periprosthetic cultures with the same organism	a- Purulence around the prosthesis, b- Acute inflammation on histopathology, c- Elevated synovial WBC count or PMN%, d- Single positive culture	One major criterion or all minor criteria
ICM on PJI 2018[18]	1- Sinus tract communicating with the prosthesis, 2- Two positive periprosthetic cultures with the same organism	*Serum biomarkers:* Elevated CRP > 10 mg/L or D-dimer > 860 ng/mL → 2 points Elevated ESR > 30 mm/h → 1 point *Synovial fluid biomarkers:* WBC count: > 10,000 cells/μL within 90 days of index surgery → 3 points > 3,000 cells/μL after 90 days of index surgery → 3 points PMN%: > 90% within 90 days of index surgery → 3 points > 80% after 90 days of index surgery → 2 points Synovial CRP > 6.9 mg/L → 1 point Positive Leukocyte Esterase Test (Grade 2 or higher) → 3 points Positive Alpha-Defensin Test (Lateral Flow or ELISA) → 3 points *Microbiological evidence:* Single positive culture from periprosthetic tissue or synovial fluid → 2 points *Histological Evidence:* > 5 neutrophils per HPF in 5 HPFs (× 400 magnification) → 3 points *Intraoperative Findings:* Purulence in the affected joint → 3 points	One major criterion or following scoring system for minor criteria: ≥ 6 points → PJI confirmed 4–5 points → Inconclusive (additional evaluation) ≤ 3 points → PJI unlikely a combination of minor criteria totaling ≥ 6 points: - Serum CRP or D-dimer: 2 points - ESR: 1 point - Synovial WBC or leukocyte esterase: 3 points - Synovial PMN%: 2 points - Synovial CRP: 1 point - Positive alpha-defensin: 3 points - Single positive culture: 2 points - Histology: 3 points - Purulence: 3 points
European Bone and Joint Infection Society (EBJIS) 2021[49]	**Definite PJI:** (if at least one of the following major criteria is met) 1- Presence of a sinus tract communicating with the prosthesis 2- Two or more cultures with the same microorganism **PJI Likely***: (*if a combination of minor criteria suggests infection) a- Periprosthetic Tissue or Synovial Fluid Markers b- WBC count: Acute infection (< 4 weeks post-op): > 10,000 cells/μL Chronic infection (> 4 weeks post-op): > 3,000 cells/μL c- PMN%: Acute infection: > 90% Chronic infection: > 80% c- *Synovial fluid biomarkers:* Positive alpha-defensin test, Positive leukocyte esterase test (grade 2 or higher) d- *Serum markers* CRP: Acute infection: > 100 mg/L Chronic infection: > 10 mg/L ESR > 30 mm/h e- *Histopathology* > 5 neutrophils per HPF in 5 HPFs (× 400 magnification) f- *Intraoperative findings* Visible purulence in the joint or surrounding tissue Single positive culture from periprosthetic tissue or synovial fluid **PJI Unlikely:** (if all of the following are absent) Sinus tract or multiple positive cultures, Elevated inflammatory markers (serum or synovial fluid), Histopathological evidence of neutrophils, Purulence		

Among the studies reviewed, ICM 2013 [48] and ICM 2018 [18] emerged as the most commonly utilized diagnostic frameworks. Notably, LE testing (grade 2 or higher) was officially included as a minor criterion in both ICM 2018 and the EBJIS 2021 guidelines [49], thus underscoring its diagnostic significance in assessing PJI. However, in studies employing ICM 2018 or EBJIS 2021, LE was not utilized as part of the gold standard for diagnosing PJI. Instead, these studies relied on histological and microbiological cultures from periprosthetic samples to confirm the presence of infection (Table 2).

### Optimal cutoff point

The diagnostic performance of LE about centrifuging all specimens and varying cut-off levels was evaluated, as shown in Table 3. When centrifuging with a cutoff of +  +, it showed pooled sensitivity of 0.899 and specificity of 0.924, a mean Youden-index of 0.823, and a DOR of 108.6. For centrifuging with a cutoff of +  +  +, showed pooled sensitivity of 0.744 and an exceptionally high specificity of 0.997 (95% CI: 0.930–1.000), with a mean Youden-index of 0.741, and a high DOR of 1068.1. In scenarios without centrifuging, a cutoff of +  + yielded a pooled sensitivity of 0.889 and specificity of 0.920, a mean Youden index of 0.809, and a DOR of 92.2. Without centrifuging and with a cutoff of +  +  +, showed pooled sensitivity of 0.877 and a specificity of 0.957, a mean Youden-index of 0.834, and a DOR of 159.2.

**Table 3:** Diagnostic values of leucocyte esterase (LE) regarding centrifuging and different cut-offs. Numbers are reported in Mean (95% confidence interval)

	**Number of studies**	**Sensitivity**	**Specificity**	**False Positive Rate (FPR)**	**Mean Youden-index**	**Diagnostic Odds Ratio (DOR)**
Centrifuging and cut-off of + +	12	0.899 (0.820–0.946)	0.924 (0.881–0.952)	0.076 (0.048–0.119)	0.823	108.6 (52.2–225.6)
Centrifuging and cut-off of + + +	6	0.744 (0.598–0.850)	0.997 (0.930–1.000)	0.003 (0.000–0.070)	0.741	1068.1 (48.9–23,329)
No centrifuging and cut-off of + +	10	0.889 (0.888–0.890)	0.920 (0.920–0.921)	0.080 (0.079–0.080)	0.809	92.2 (91.2–93.2)
No centrifuging and cut-off of + + +	7	0.877 (0.769–0.939)	0.957 (0.922–0.977)	0.043 (0.023–0.078)	0.834	159.2 (69.5–364.4)

### Direct comparison of LE to other biomarkers

Direct comparisons of other markers’ sensitivity and specificity to LE’s sensitivity and specificity are available in Table 4. Their respective sROC curves are presented in the Supplementary Information.

**Table 4:** Direct comparison of LE to other markers. Numbers are reported in Mean (95% confidence interval)

	**Number of studies**	**Another diagnostic test**					**LE**				
		**Sensitivity**	**Specificity**	FPR*	DOR*	**Mean Youden-index**	**Sensitivity**	**Specificity**	**FPR**	**DOR**	**Mean Youden-index**
PMN	7	0.855 (0.780–0.907)	0.883 (0.803–0.933)	0.117 (0.067–0.197)	44.4 (25.7–76.5)	0.738	0.750 (0.700–0.795)	0.919 (0.890–0.941)	0.081 (0.059–0.110)	34.0 (24.7–46.7)	0.669
WCC	8	0.839 (0.773–0.889)	0.913 (0.862–0.946)	0.087 (0.054–0.138)	54.7 (29.6–101.3)	0.752	0.777 (0.718–0.826)	0.907 (0.874–0.933)	0.093 (0.067–0.126)	34.0 (25.1–46.1)	0.684
ESR	6	0.814 (0.741–0.870)	0.797 (0.712–0.861)	0.203 (0.139–0.288)	17.1 (12.2–24.2)	0.611	0.769 (0.717–0.813)	0.913 (0.894–0.930)	0.087 (0.070–0.106)	35.0 (24.6–49.9)	0.682
Serum CRP	8	0.835 (0.743–0.899)	0.793 (0.699–0.863)	0.207 (0.137–0.301)	19.3 (13.0–28.6)	0.628	0.747 (0.698–0.790)	0.926 (0.895–0.949)	0.074 (0.051–0.105)	37.1 (26.2–52.5)	0.673
Alpha-Defensin	9	0.826 (0.771–0.870)	0.967 (0.930–0.985)	0.033 (0.015–0.070)	139.1 (63.4–305.1)	0.793	0.770 (0.716–0.816)	0.934 (0.901–0.956)	0.066 (0.044–0.099)	47.0 (29.0–76.1)	0.704
Synovial CRP	4	0.844 (0.782–0.890)	0.917 (0.874–0.946)	0.083 (0.054–0.126)	59.6 (33.3–106.7)	0.761	0.854 (0.739–0.924)	0.944 (0.899–0.970)	0.056 (0.030–0.101)	99.4 (30.8–320.8)	0.798
D-dimer	2	0.748 (0.562–0.872)	0.802 (0.195–0.985)	0.198 (0.015–0.805)	11.9 (1.3–106.4)	0.550	0.767 (0.683–0.834)	0.893 (0.844–0.928)	0.107 (0.072–0.156)	27.4 (14.9–50.2)	0.660

^*^FPR: False Positive Rate, DOR: Diagnostic Odds Ratio

PMN%, WCC, and AD showed a slightly better DOR (44.4, 54.7, 139.1 vs 34.0, 34.0, 47.0; respectively) and Youden-index (0.738, 0.752, 0.793 vs 0.669, 0.684, 0.704; respectively) compared to LE. ESR, serum CRP, synovial CRP, and D-dimer showed lower DOR (17.1, 19.3, 59.6, 11.9 vs 35.0, 37.1, 99.4, 27.4; respectively) and Youden-index (0.611, 0.628, 0.761, 0.550 vs 0.682, 0.673, 0.798, 0.660; respectively) compared to LE.

### Quality assessment

The results of QUADAS-2 assessments for each included study are shown in Table 5. In each critical domain, the proportion of high-risk studies was less than 5%, indicating that the included studies’ quality was good.

**Table 5:**
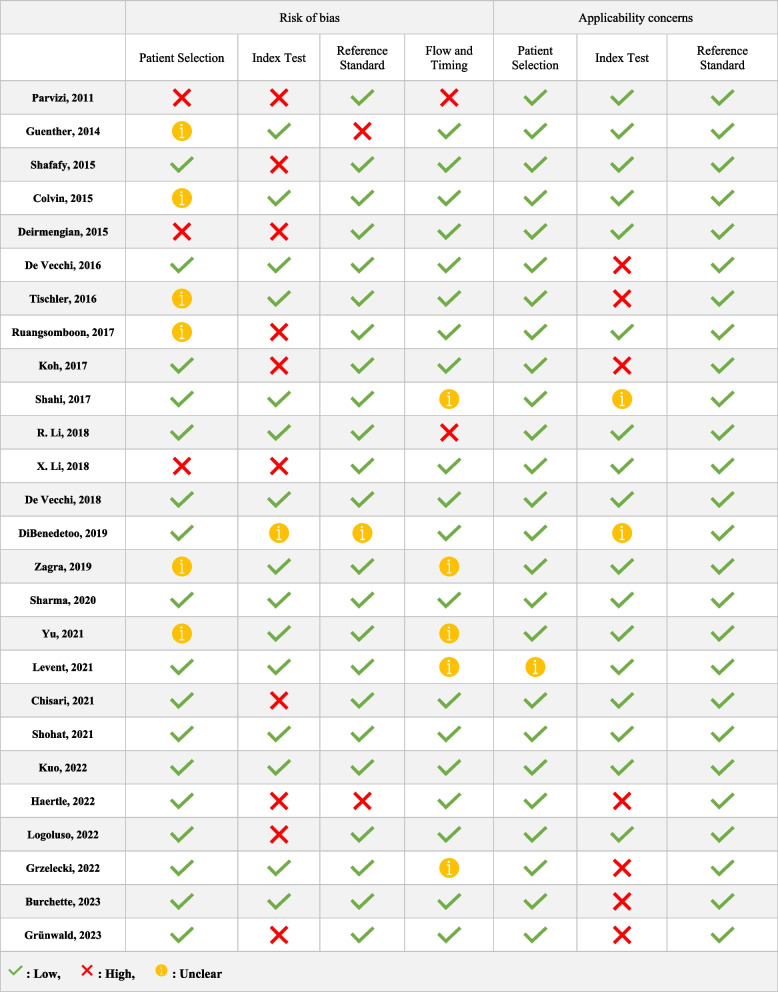
Quality assessment of included studies. [10, 11, 20–26, 29, 31–46]

## Discussion

This study aimed to determine the optimal cut-off point for LE in diagnosing PJI, both with and without centrifugation. Additionally, we compared the diagnostic performance of LE with other established markers. Our results indicated that without centrifugation, a cut-off point of 3 + for LE yielded the highest mean Youden Index of 0.834. This cut-off demonstrated a sensitivity of 0.877 and a specificity of 0.957. However, when the centrifugation was applied, a cut-off point of 2 + for LE provided the best mean Youden Index of 0.823. This cut-off showed a sensitivity of 0.899 and a specificity of 0.924. The DOR for LE without centrifugation at a 3 + cut-off was 159.2 (95% CI, 69.5 to 364.4), and with centrifugation at a 2 + cut-off, it was 108.6 (95% CI, 52.2 to 225.6). In clinical practice, our findings support adopting a 3 + cut-off for LE testing on uncentrifuged synovial fluid and a 2 + cut-off after centrifugation to maximize diagnostic accuracy. These thresholds yield sensitivity and specificity values that surpass those of other cut-offs, facilitating more reliable point-of-care decision-making.

### Clinical implications

Based on the minor criteria of the ICM 2018 and EIJBS 2021 [18, 49], a 2 + reading of LE is assigned a score of 3 for diagnosing PJI. However, our analysis indicates that a consistent 2 + reading is only sometimes reliable across different methodologies. Of note, one potential drawback of LE testing is that contamination with blood or other particles in the synovial fluid may disrupt the color change observed on the urinalysis strip [51]. Consequently, centrifugation for at least 2 min may be necessary for accurate result interpretation [52]. Our results showed that post-centrifugation results tend to be slightly lighter than pre-centrifugation samples. When centrifugation is performed, grades 2 + and 3 + should be considered positive for infection to achieve the highest diagnostic accuracy. In contrast, without centrifugation, a grade 3 + reading is more accurate for diagnosing PJI.

### Impact of centrifugation

In several studies, the LE strip test results could not be accurately read without centrifugation due to color disturbance caused by blood contamination or other particles (Table 1). Therefore, the blood-synovial fluid mixture was transferred from the syringe to a standard centrifuge tube immediately and then subjected to centrifugation. The centrifugation settings varied across studies, ranging from 3000 to 10,000 revolutions per minute (rpm) [21, 43], and the duration of centrifugation also differed. After centrifugation, one drop of the clarified fluid was placed on the LE strip, and the results were read after approximately 60–120 s of reaction time.

A study by Li et al. investigated the optimal cut-off point for LE both before and after centrifugation [24]. They found that centrifugation significantly lightened the color of almost every sample. They reported that, before centrifugation, using a grade 3 + as the positive criterion was deemed more appropriate. However, after centrifugation, grades 2 + and 3 + were more suitable as the positive criteria [24]. Another recent study by Yu et al. performed the LE test both before and after centrifugation, using different thresholds for result interpretation [22]. When applying a threshold of 500 (equivalent to 3 +) after centrifugation, the sensitivity and specificity of the LE strip test were reported to be 75.4% and 95.4%, respectively, with an area under the curve (AUC) of 0.854. However, when both 250 and 500 were considered positive (equivalent to 2 +), the sensitivity improved to 80.0%, while specificity remained stable at 95.4%. This adjustment also resulted in an improved AUC of 0.877 [22]. These findings are consistent with our results, which indicated that a cut-off point of 3 + for LE provided a better mean Youden Index before centrifugation. In contrast, a cut-off point of 2 + and 3 + showed superior results after centrifugation.

Our analysis further revealed that centrifugation and a cut-off point of 3 + for LE testing resulted in the highest specificity of 0.997. The DOR of 1068.1 (95% CI, 48.9 to 23,329) for cut-off point 3 +, indicated that a positive test result would almost certainly confirm the presence of PJI. Centrifugation of bloody joint aspirates significantly enhances the diagnostic utility of the LE enzyme test for PJI. The efficiency of this method is further highlighted by its rapid sample processing, with results available within minutes [53]. The low cost of a minicentrifuge and chemical test strips, combined with high diagnostic accuracy, justifies using LE enzyme testing as an effective and efficient method for diagnosing PJI [53].

### Comparison with other biomarkers

Most medical professionals may use ESR and serum CRP as the initial screening method since this method is cost-effective and non-invasive. In contrast to the findings of Carli et al., we found that ESR (80.8% vs. 76.2%) and serum CRP (85.5% vs. 79.8%) have higher sensitivity than LE [54]. Similar to their findings, we found that ESR (80.5% vs. 91.1%) and serum CRP (81.5% vs. 92.7%) have lower specificity than LE [54]. We also found that LE has higher DOR than ESR (32.9 vs. 17.3) and serum CRP (50.4 vs. 25.9). This shows that LE, with its superior specificity, can help confirm cases in combination with ESR and CRP.

PMN%, WCC, and LE share a similar underlying mechanism in diagnosing PJI, as they all reflect inflammatory responses within the synovial fluid [55]. Consequently, it is unsurprising that these markers demonstrate minimal differences in terms of sensitivity and specificity [17]. PMNs serve as the body's primary defense against infection and initiate the inflammatory response [56]. They become activated at the site of infection and subsequently release LE [34]. Thus, the PMN% is a component of WCC, and the LE test is a derivative of both WCC and PMN%. Wang et al. have discussed that the LE test exhibits a significant correlation with synovial WCC and PMN% in diagnosing PJI [16]. Our study has shown that WCC is more sensitive (85.1% vs. 79.1%) and specific (92.3% vs. 90.1%) than the LE test. Additionally, we observed that the PMN% had a higher sensitivity (86.6% vs. 77%) but lower specificity (85.2% vs. 90.9%) compared to the LE test. However, LE and PMN% exhibited a similar DOR (37 vs. 33.4). A meta-analysis conducted by Qu et al. analyzed 15 articles and reported results that closely align with our study [57].

In synovial fluid analysis, the chronicity of PJI is pivotal in establishing diagnostic thresholds. For acute PJI, occurring within 6 weeks postoperatively, the synovial WCC is recommended to exceed 10,000 cells/μL, with PMN% greater than 90% [17]. In contrast, for chronic PJI, defined as occurring beyond 6 weeks post-surgery, these thresholds are significantly lowered to over 3,000 cells/μL and more than 80%, respectively [17] (Table 2). However, unlike WCC and PMN%, which fluctuate based on the infection’s phase, the LE enzyme maintains a consistent threshold for both acute and chronic PJI, as supported by ICM 2018 and EBJIS 2021 (defining a grade of 2 + as positive) (Table 2). This stability is attributed to the mechanism of LE detection, which strongly binds to receptors at the infection site [17, 58].

During infection, AD is produced by neutrophils and is elevated in synovial fluid [59]. Our study revealed that AD is more sensitive (81.7% vs. 78.5%) and more specific than LE (95.7% vs. 93.3%). A study by Vale et al. in 2023 also demonstrated that AD has greater diagnostic accuracy than LE [60], which aligns with our findings. However, a meta-analysis by Chen et al. of 28 studies reported nearly identical results for both tests [61]. They included 16 articles for AD and 12 papers for LE in their final analysis. Chen et al. pooled the results of all LE and AD studies in the literature. In contrast, our study pooled data from studies that used both LE and AD on the same samples, providing a more accurate and direct comparison of these biomarkers.

D-dimer is a specific marker of the fibrinolysis process, primarily reflecting fibrinolytic function [62]. It has been suggested as a prognostic tool for systemic sepsis [63]. In the context of PJI, D-dimer levels were significantly higher in PJI cases [64]. However, previous studies have demonstrated that D-dimer has poor diagnostic accuracy for PJI [64, 65]. Our analysis corroborates this, showing that D-dimer has lower sensitivity and specificity than LE, with a notably low DOR of 11.9.

#### Limitations

We observed considerable variability among the dipstick tests used for LE assessment. The tests differed in the number of blocks; some included four blocks (e.g., Chemstrip 7, Roche), while others contained five blocks (e.g., Multistix 8, Siemens). Furthermore, the grading of LE levels varied both within and across tests. For instance, the Multistix 8 (Siemens) classified results as negative, 15, 70, 125, and 500, while Aution Sticks 10PA (Arkray) utilized negative, 25, 75, 250, and 500. Even tests with the same block count and grading systems provided differing results; for example, Chemstrip 7 and Combur 2 (both from Roche Diagnostics) reported results as Neg, Trace, 1 +, 2 +, and Neg, 1 +, 2 +, 3 +, respectively. To address these discrepancies, we standardized the LE results across studies to match the reporting format of the Combur 2 dipstick, aiming for the most consistent data comparison possible. However, due to intrinsic differences in grading and reporting, achieving complete harmonization of the test results proved unfeasible, highlighting a limitation in our analysis.

Secondly, the classification of PJI chronicity was inconsistent among the studies included in the analysis. While most studies concentrated on acute PJI (defined as occurring within ≤ 6 weeks postoperatively), some also incorporated patients with chronic PJI (occurring > 6 weeks postoperatively). Some studies did not indicate whether the infections were acute or chronic. As previously mentioned, this variability does not affect the interpretation of the LE test, as its diagnostic threshold remains constant regardless of the infection’s chronicity. However, differences in chronicity may have impacted the diagnostic performance of other synovial markers, such as WCC and PMN%, which possess distinct cutoff values based on whether the infection is acute or chronic. This heterogeneity and the insufficient reporting of chronicity in certain studies may have contributed to variability in the sensitivity and specificity estimates for these biomarkers.

## Conclusion

In conclusion, the LE test is a highly effective diagnostic tool for PJI, particularly when centrifugation is utilized to achieve accurate readings. Adopting a 3+ cut-off point without centrifugation and a 2+ cut-off point with centrifugation can optimize diagnostic accuracy, thereby improving patient outcomes.

## Supplementary Information

Supplementary Material 1.

## Data Availability

No datasets were generated or analysed during the current study.
