# Supplementary material for: What is the best cutoff point of leukocyte esterase for diagnosis of periprosthetic joint infections? a systematic review and meta-analysis
Source: Arthroplasty. 2025 Aug 5;7:41. doi: 10.1186/s42836-025-00325-y (PMC12323279; doi:10.1186/s42836-025-00325-y)

sROC curve of PMN:
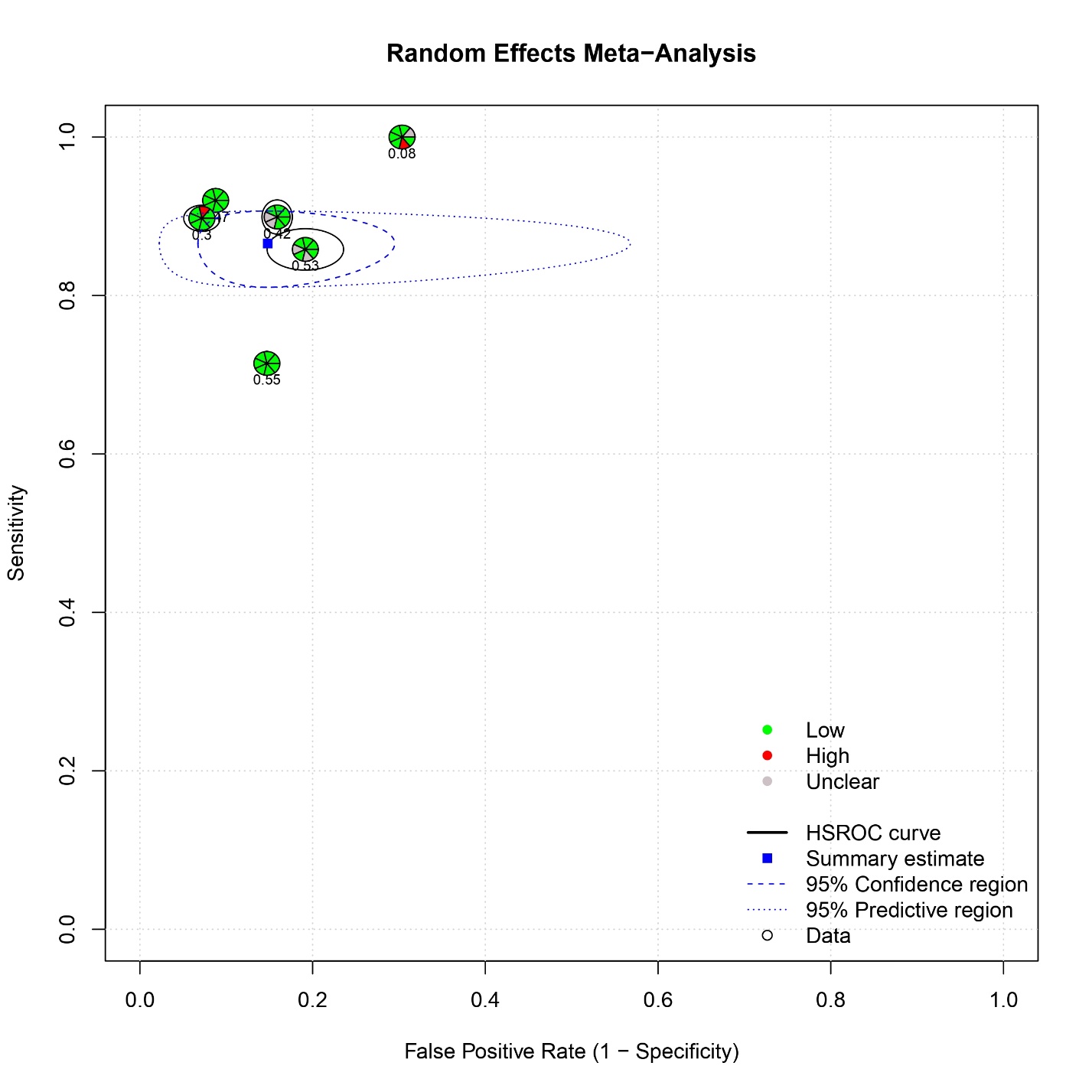


sROC curve of LE in studies that reported PMN:


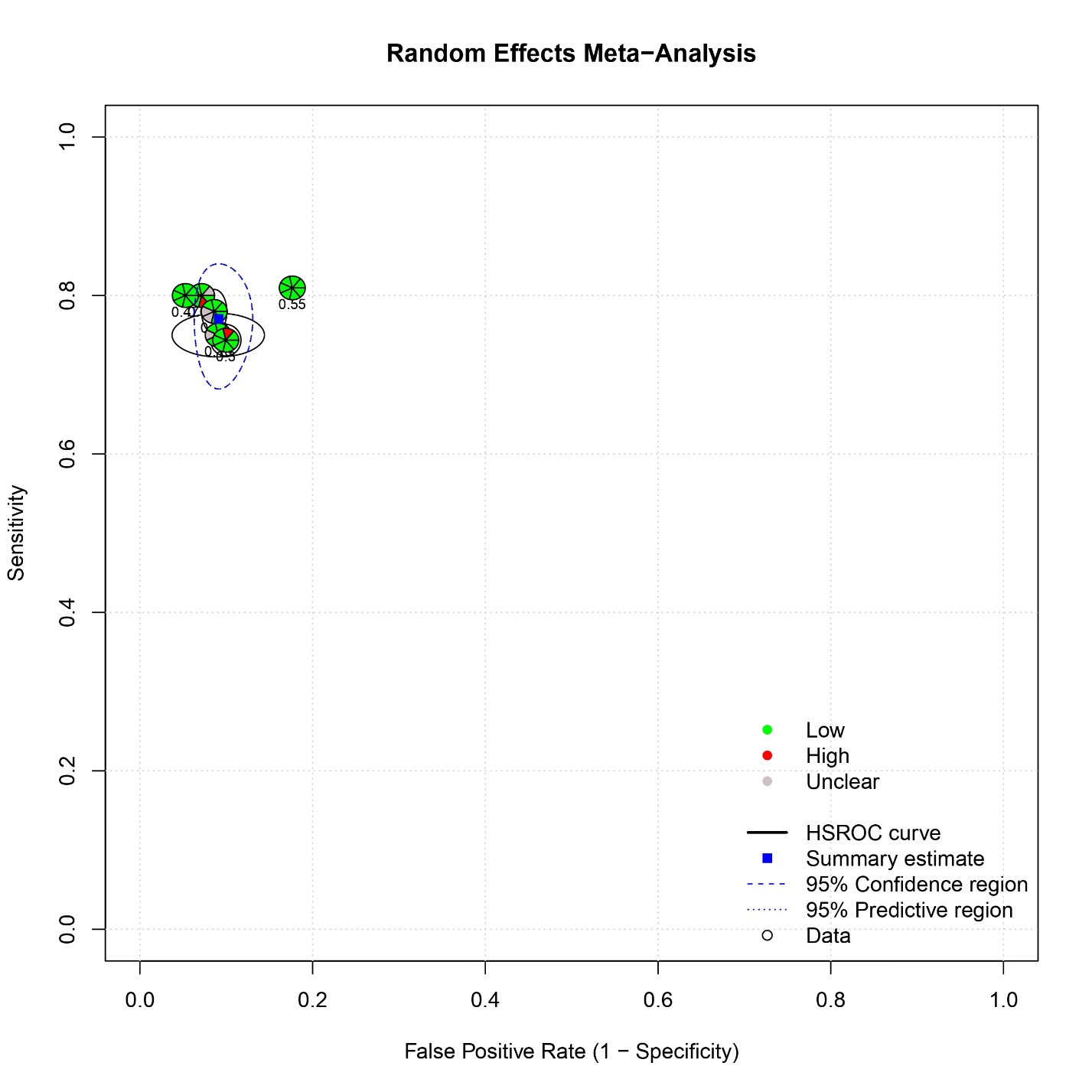


sROC curve of WBC:


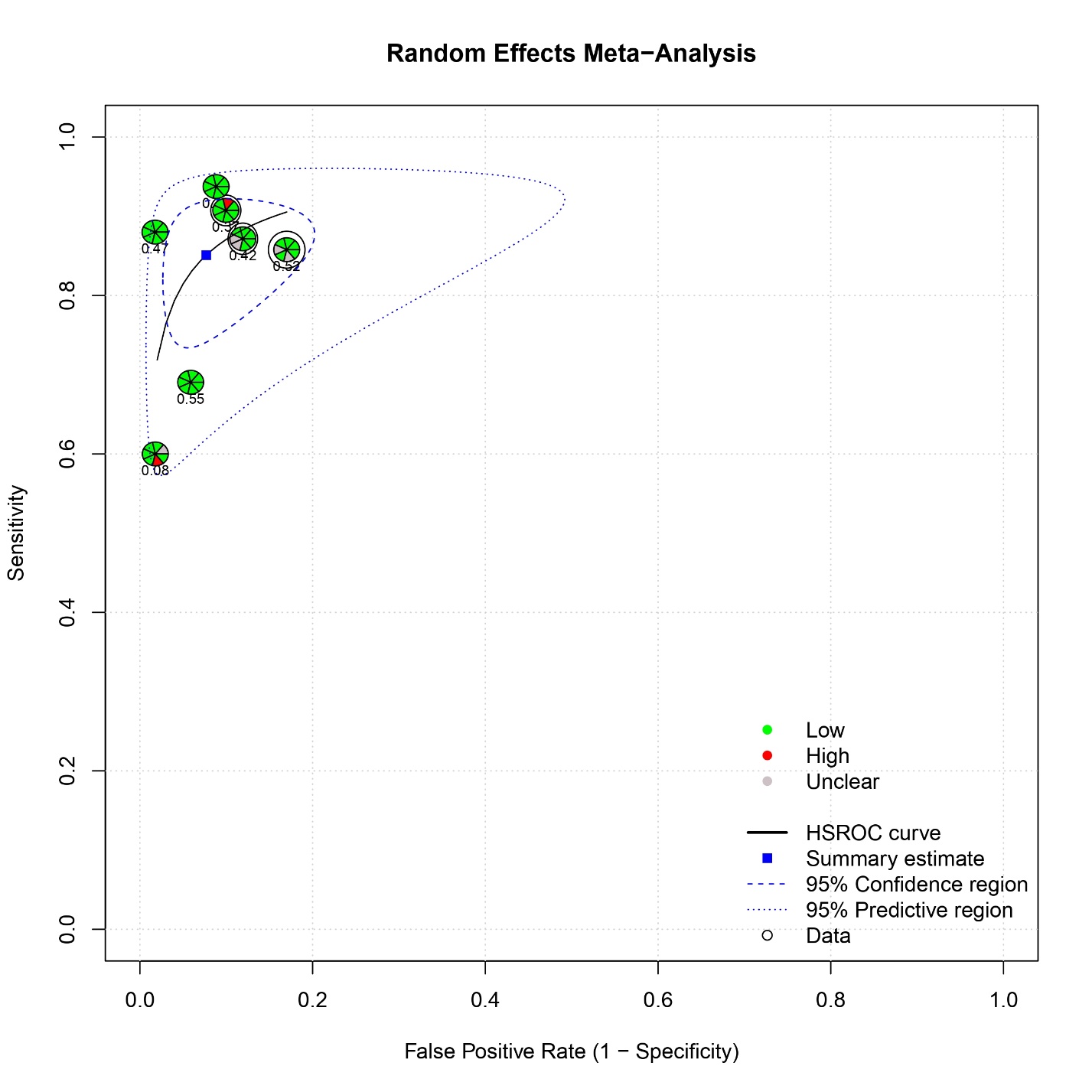


sROC curve of LE in studies that reported WBC:


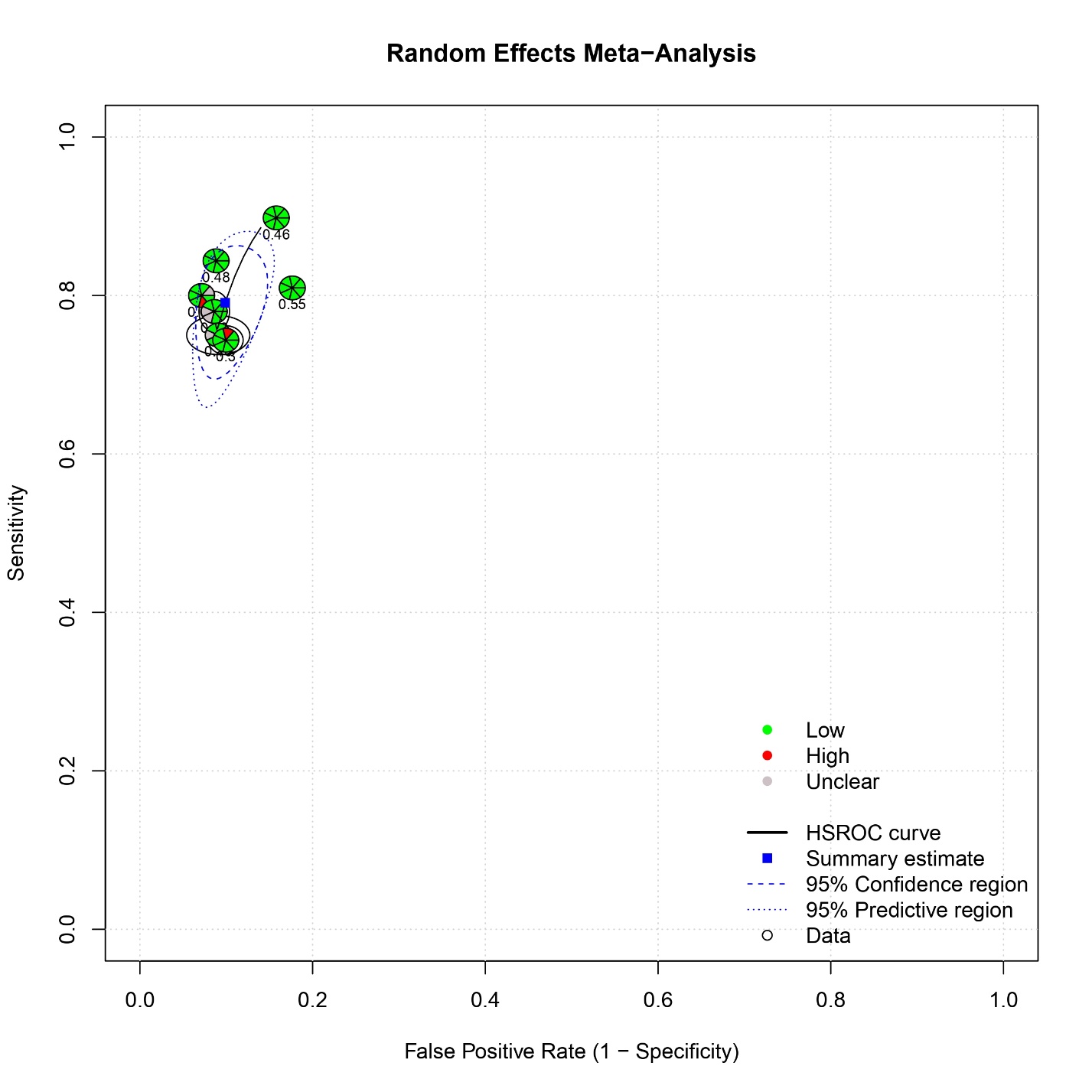


sROC curve of Alpha-Defensin:


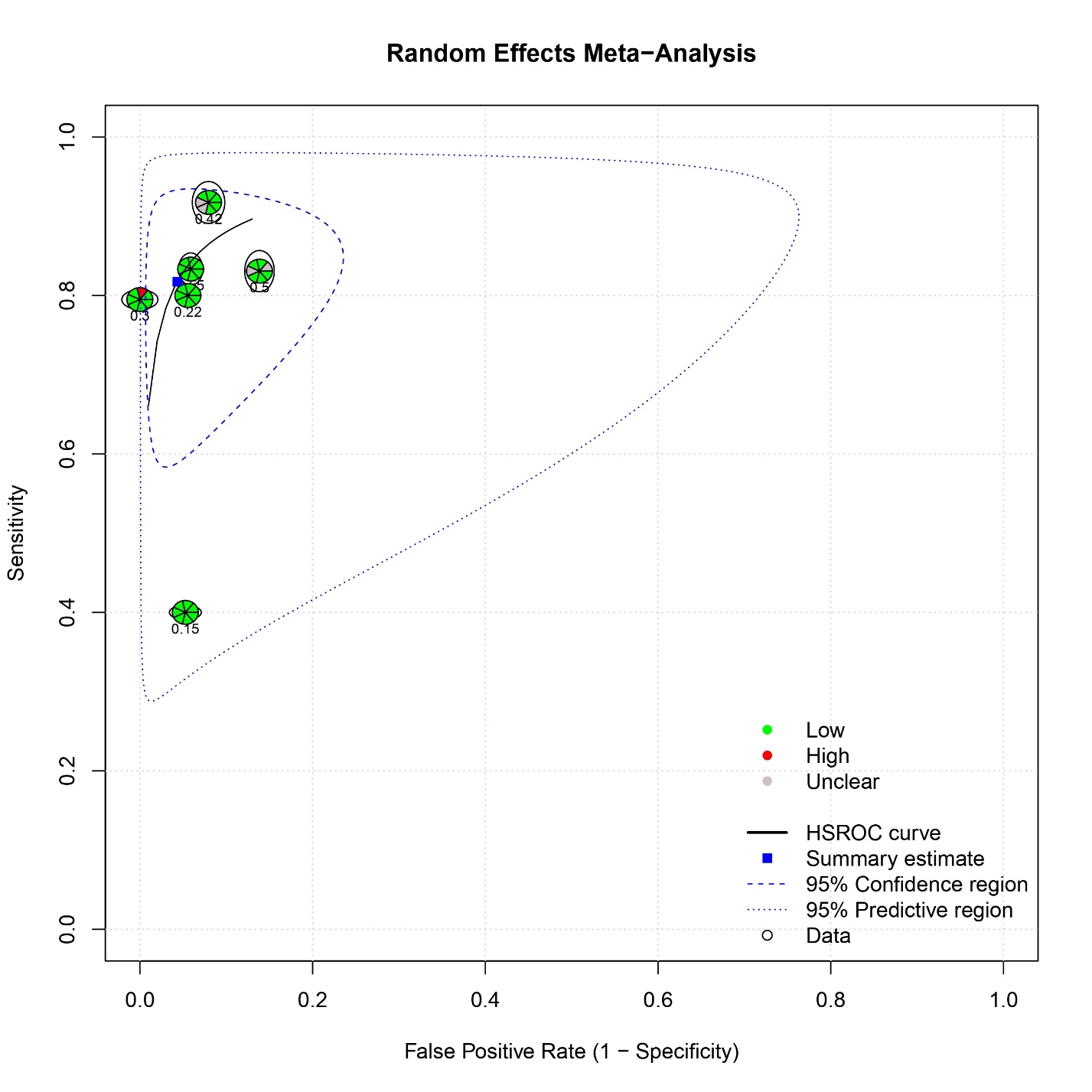


sROC curve of LE in studies that reported Alpha-Defensin:


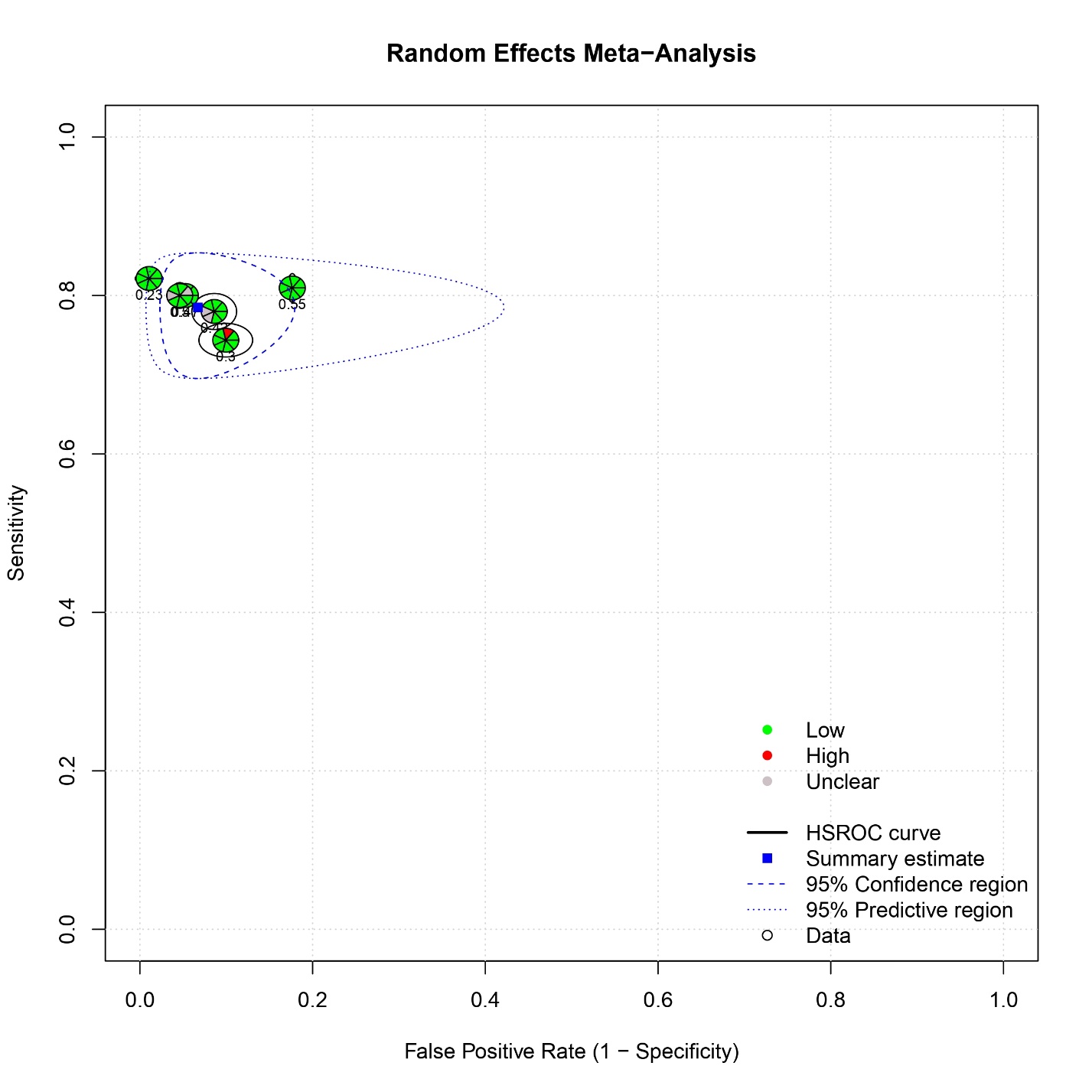


sROC curve of CRP:


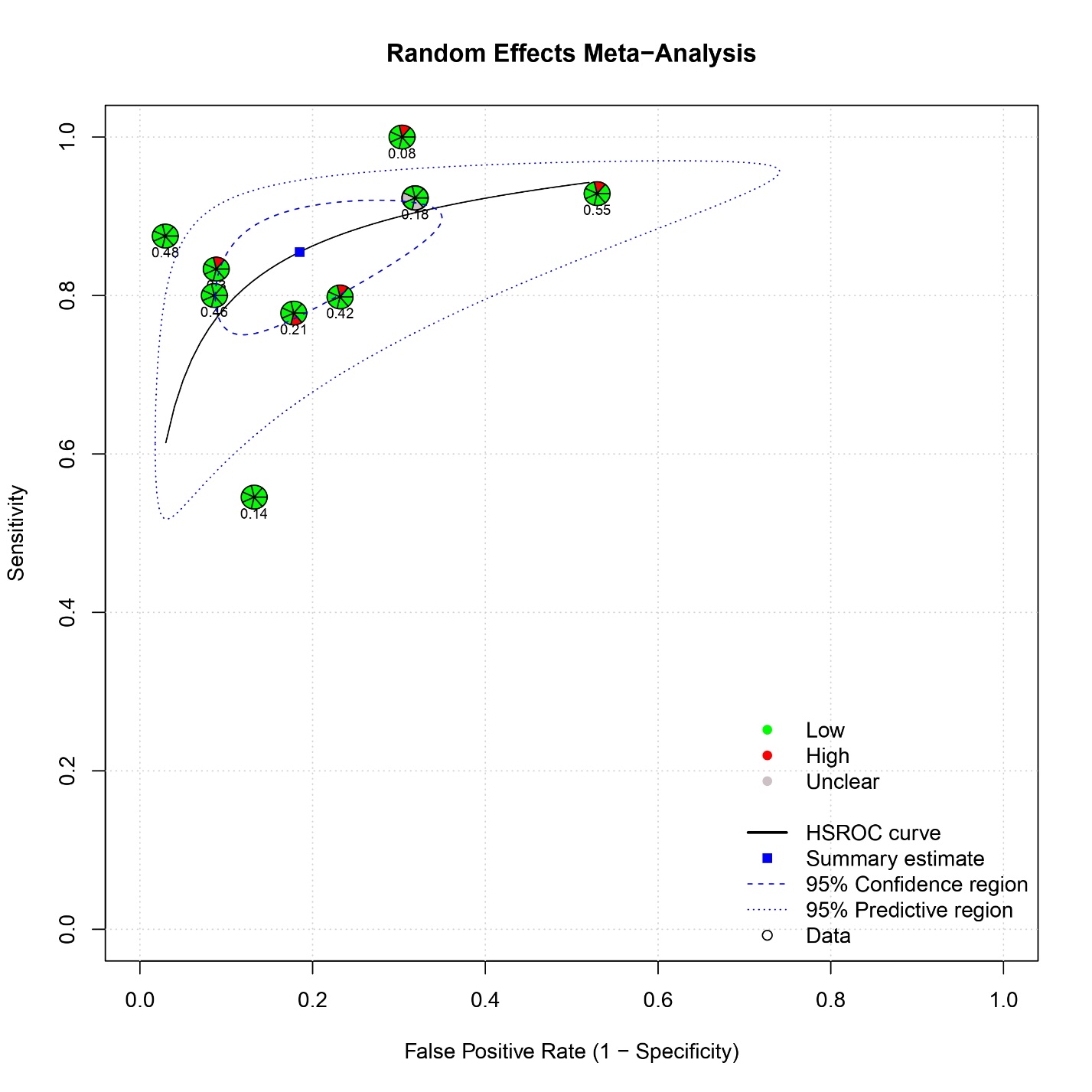


sROC curve of LE in studies that reported CRP:


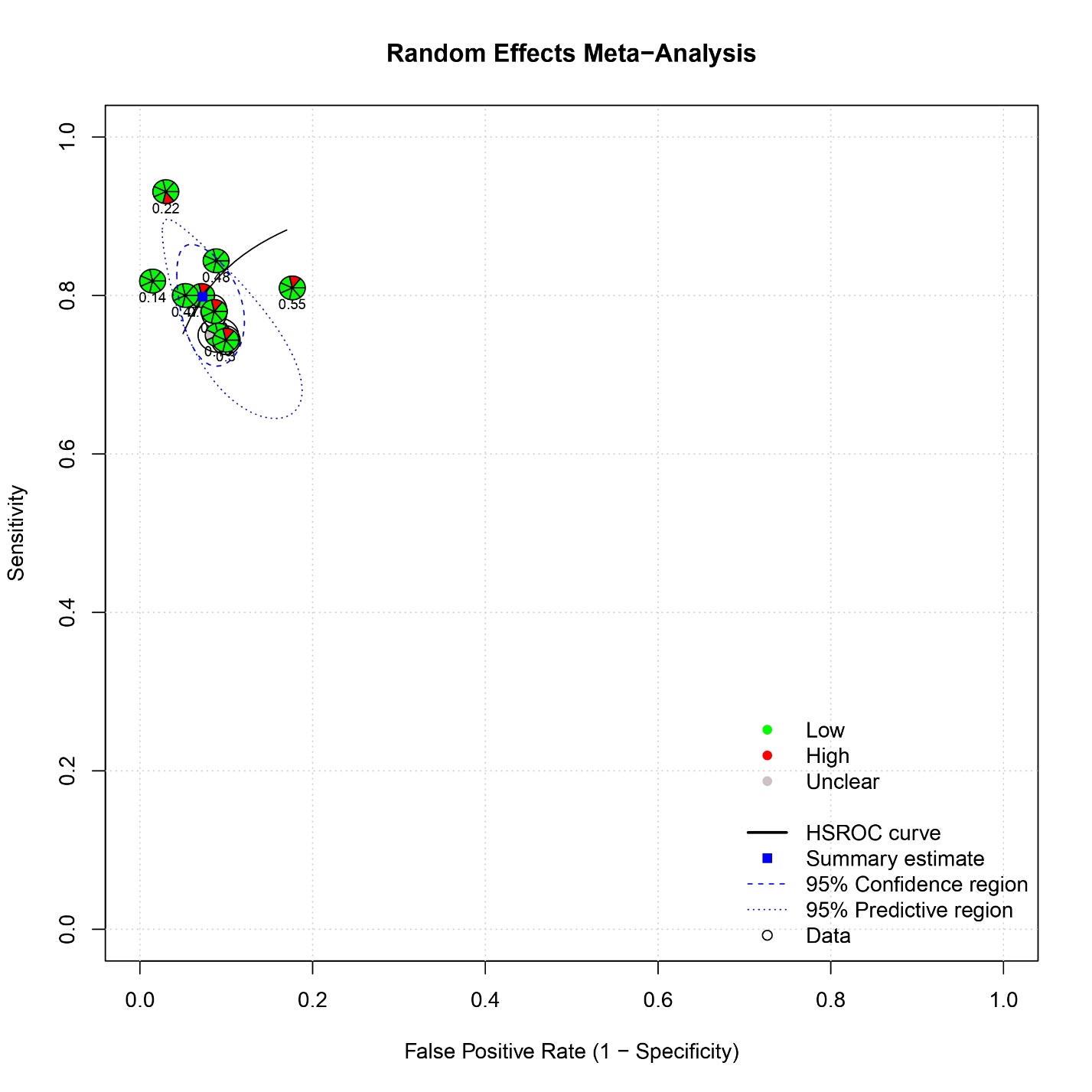


sROC curve of ESR:


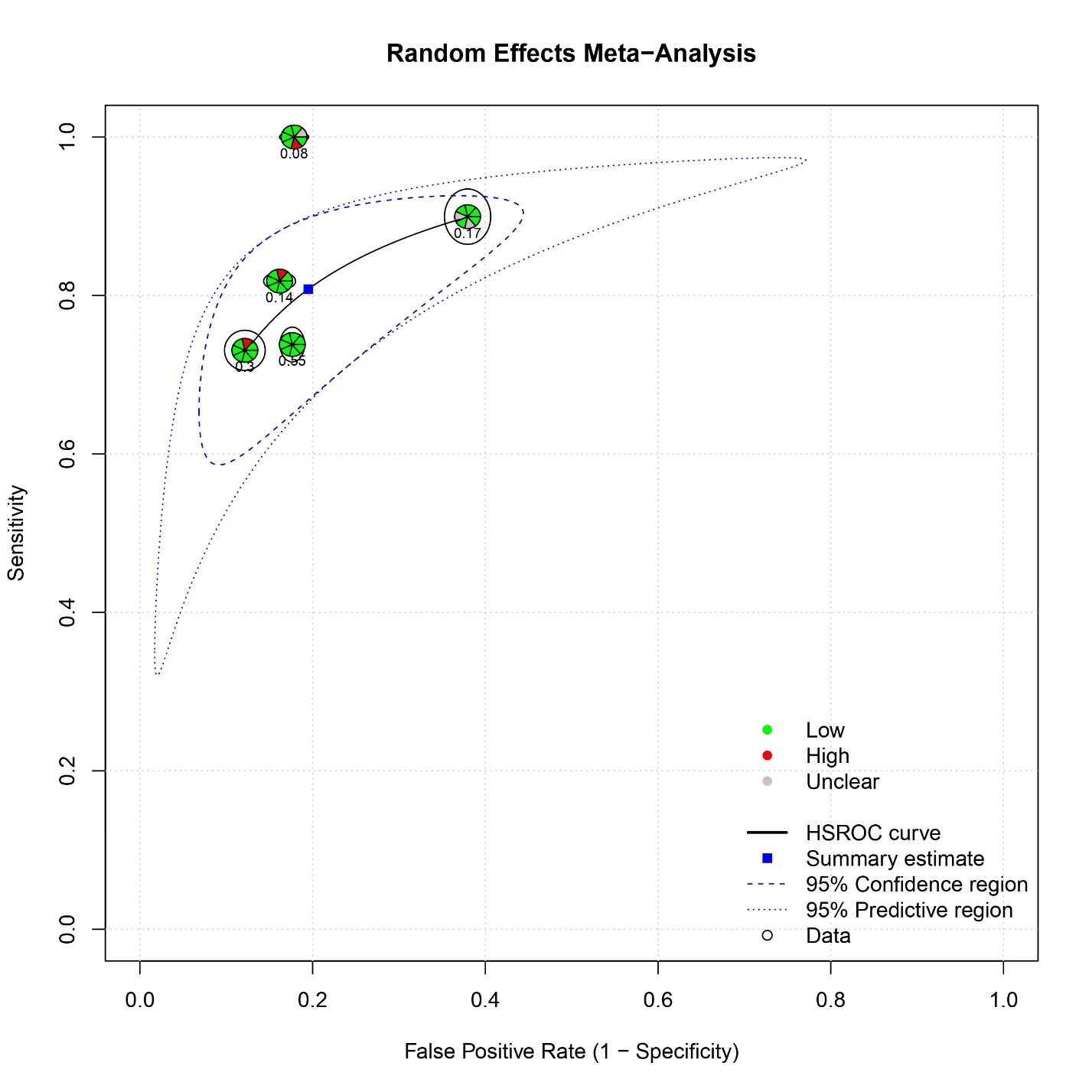


sROC curve of LE in studies that reported ESR:


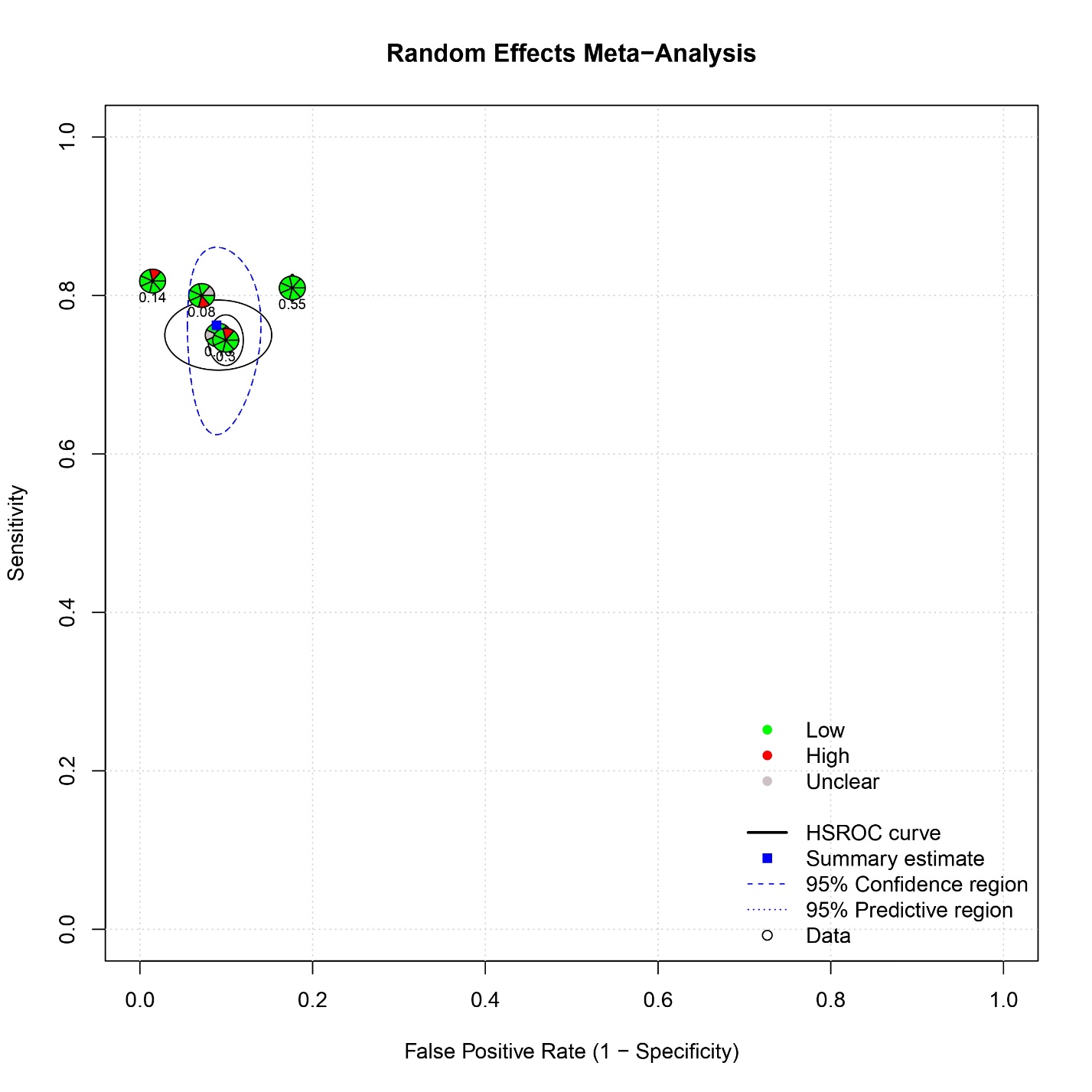

Supplement: Supplementary file 1 — Supplementary Material 1. [file 42836_2025_325_MOESM1_ESM.docx]
